# Sex differences in memory impairments after traumatic brain injury: mechanisms and models

**DOI:** 10.3389/fnbeh.2026.1874990

**Published:** 2026-07-31

**Authors:** Isabella K. Robson, Clement Hamani

**Affiliations:** 1 Sunnybrook Research Institute, Toronto, ON, Canada; 2 Institute of Medical Science, School of Graduate Studies, University of Toronto, Toronto, ON, Canada; 3 Division of Neurosurgery, Sunnybrook Health Sciences Centre, University of Toronto, Toronto, ON, Canada; 4 Hurvitz Brain Sciences Program, Harquail Center for Neuromodulation, Toronto, ON, Canada

**Keywords:** females, males, memory, preclinical models, sex, traumatic brain injury

## Abstract

Traumatic brain injury (TBI) is a major cause of long-term cognitive impairment, with deficits in episodic memory among the most common and disabling consequences. Although biological sex is increasingly recognized as an important determinant of TBI outcomes, its role in shaping post-injury memory impairment remains incompletely understood. Findings from both clinical and preclinical studies are often inconsistent, and sex is frequently underrepresented in experimental designs. Clinical studies generally indicate that women experience worse cognitive outcomes after TBI, including more severe and persistent memory deficits and greater affective burden. In contrast, preclinical findings are more variable, with sex differences emerging in a domain-specific and context-dependent manner. Early animal studies often reported no significant sex differences, particularly in spatial memory tasks. More recent work reveals that males tend to show earlier and more widespread spatial learning impairments and greater vulnerability to anterograde memory deficits, while females more commonly exhibit delayed but persistent deficits in memory consolidation and retention. These behavioral differences are accompanied by sex-specific mechanisms. Altogether, sex differences in memory outcomes following TBI are complex, context-dependent, and shaped by both biological and methodological factors. Greater incorporation of sex as a biological variable in both clinical and preclinical research is essential. Moving toward a precision medicine framework, accounting for sex as a variable in therapeutic target selection and trial design will be important for optimizing cognitive recovery after TBI.

## Introduction

Traumatic brain injury (TBI) is a heterogeneous disorder defined as an alteration in brain function caused by biomechanical forces applied to the head or neck (Maas et al., 2017). Clinically, TBI can present with focal contusions, hemorrhages, hematomas, penetrating wounds, diffuse injuries, or concussions (Wang et al., 2018). Although ∼80% of cases are characterized by mild injury (mTBI) (Huang et al., 2024), moderate-to-severe TBI (msTBI) is associated with increased mortality (United States Department of Health and Human Services [US DHHS] et al., 2024), reduced quality of life (Centers for Disease Control and Prevention (U.S.) et al., 2021), and a high proportion of long-term disability (Jourdan et al., 2016). Long-term sequelae may include somatic complaints (e.g., fatigue, headaches, and pain) and cognitive symptoms. Among the latter, episodic memory deficits are particularly common, with approximately 68% of msTBI patients reporting some degree of impairment 4 years post-injury (Jourdan et al., 2016). Beyond memory-specific effects, individuals with msTBI also face a higher long-term risk of cognitive decline, neurodegenerative disease, poor health, and increased personal care needs (Kumar et al., 2020; Sigurdardottir et al., 2020).

Despite the prevalence and severity of these outcomes, biological sex remains a critically underexplored determinant of post-TBI cognitive recovery. This mini review examines the association between sex, the incidence and outcome following TBI, as well as underlying mechanisms of memory impairment, integrating clinical and preclinical evidence.

## Sex differences in TBI: incidence and outcomes

The incidence of TBI is approximately 40% higher in men, partly reflecting historical gender differences in exposure to high-risk activities, behaviors, and occupations (Faul et al., 2010). However, as societal roles evolve, an increasing number of cases are being identified in women (Giza et al., 2013; Kutcher and Giza, 2014; Ledreux et al., 2020), with particular vulnerability in older adults (Faul et al., 2010; Ma et al., 2019). Sex-related differences also exist in mechanisms of injury. Women are more likely to sustain TBI through assault or violence, whereas men more often experience work-related injuries from falls or motor vehicle collisions (Chang et al., 2014; Kwako et al., 2011). Importantly, the reported incidence of TBI is likely underestimated in women due to substantial underreporting of intimate partner violence (IPV), a highly gendered context in which most victims are women (Biegon, 2021). Given that 60%–90% of IPV survivors sustain facial or head injuries and only ∼20% receive treatment (Monahan and O’Leary, 1999; St. Ivany and Schminkey, 2016), the true magnitude of sex differences in TBI incidence are still unclear.

Another key and actively debated issue is whether outcomes after TBI differ by sex. Historically, women have been thought to be more likely to die following TBI (Kraus et al., 2000). While a recent meta-analysis reported a higher female mortality risk after msTBI (Breeding et al., 2024), the broader literature remains controversial, with findings varying by injury severity, trauma type, and menopausal or hormonal status (Berry et al., 2009; Gupte et al., 2019; Ley et al., 2013). Similar variability has been reported for morbidity. Although women have been suggested to experience more severe disability after TBI (Howell and Griesbach, 2024; Kraus et al., 2000), results are non-uniform across cohorts and outcome measures.

Sex differences have also been observed in post-TBI symptom severity and recovery. In a scoping review of clinical studies, 47% found worse outcomes in women, 23% reported worse outcomes in men, and 30% identified no sex differences or mixed results (Gupte et al., 2019). Overall, women tend to experience poorer clinical outcomes, including higher rates of headaches, dizziness, insomnia, and amnesia (Colantonio et al., 2010; Farace and Alves, 2000). They also often have longer hospital stays and delayed return to work (Colantonio et al., 2010; Farace and Alves, 2000). After mTBI, women show greater cognitive decline (Broshek et al., 2005), poorer reaction time (Chiang Colvin et al., 2009), and worse post-concussive symptom scores (Bazarian et al., 2010). In addition, women present more depressive symptoms, greater perceived stress and pain, lower quality of life, and more prominent memory and cognitive deficits (Bay et al., 2009; Covassin and Bay, 2012; Howell and Griesbach, 2024). Notably, women also tend to experience decreased REM sleep following TBI, which is associated with reduced episodic memory performance (Howell and Griesbach, 2024). Consistent with these patterns, a large cohort study (>15,500 patients) found that women had a greater overall symptom burden, higher pain intensity, and longer recovery times than men (Arachchi et al., 2026). Together, these findings suggest that women may experience a more severe and prolonged recovery trajectory after TBI, particularly in cognitive and neurosensory domains.

## Sex differences in the pathophysiology of TBI

Gonadal sex hormones play diverse roles in homeostasis and disease (Vegeto et al., 2020) and are widely considered important modulators of TBI pathophysiology (Gölz et al., 2019). Estrogen is synthesized in both female and male brains and has been implicated in synaptic plasticity and neuroprotection (Arevalo et al., 2015). Proposed neuroprotective actions include preservation of autoregulatory function, reduced excitotoxicity, antioxidant effects, reduced amyloid-β production and neurotoxicity, increased expression of anti-apoptotic factors, and activation of mitogen-activated protein kinase (MAPK) pathways (Roof and Hall, 2000). In rodent TBI models, intact females with regular estrous cycles often show reduced susceptibility compared with males and ovariectomized females (Bao et al., 2011; Day et al., 2013; Lu et al., 2018; Maghool et al., 2013). In humans, menopause-associated estrogen decline is linked to an increased risk of cognitive impairment and neurodegenerative disorders (Scott et al., 2012). Progesterone, another hormone present in both sexes, is also well-established as neuroprotective. It can attenuate neural injury by activating MAPK/ERK and Akt signaling pathways and upregulating brain-derived neurotrophic factor (BDNF) (Brinton et al., 2008). In rodent models, exogenous progesterone reduces cerebral edema (Roof et al., 1992; VanLandingham et al., 2006; Wright et al., 2001), necrotic damage and cell loss (Robertson et al., 2006; Roof et al., 1994; Shear et al., 2002; VanLandingham et al., 2006), decreases reactive gliosis and inflammation (Robertson et al., 2006; VanLandingham et al., 2006), regulates anti-apoptotic gene expression (Yao et al., 2005), and improves cognitive outcomes (Roof et al., 1994; Shear et al., 2002). In humans, a randomized controlled trial reported a potential benefit for intravenous progesterone following msTBI (Wright et al., 2007). Hormonal fluctuations across the menstrual/estrous cycle may contribute to variability in female outcomes.

In addition to a direct hormonal effect on injury, sex differences have also been reported in neuroinflammatory responses and oxidative stress. In male mice, TBI is associated with a higher microglial and macrophage activation, COX-2 expression, enhanced astrogliosis, apoptotic cell death, and more pronounced oxidative stress responses compared to females (Bakay et al., 1986; Günther et al., 2015; Tapp et al., 2023; Villapol et al., 2017; Wagner et al., 2004a). In the clinic, women have shown reduced oxidative damage (Wagner et al., 2004a) and a significantly lower lactate/pyruvate ratio, indicating less ischemic brain hypoxia (Sahuquillo et al., 2014).

Neuroplasticity is well-known to play a crucial role in determining rehabilitation outcomes following TBI (Zotey et al., 2023) and is driven in part by various sex-specific signaling mechanisms. In the hippocampus, spine density peaks in proestrus, when estrogen and progesterone levels are highest, and declines throughout later stages (Hyer et al., 2018). Females typically have twice the spine density as males, with comparable sex numbers reported after ovariectomy (Hyer et al., 2018). Notably, increases in CA1 spine density in ovariectomized mice treated with low-dose estrogen correlated with improved learning (Phan et al., 2012). In addition, females have increased levels of estrogen receptors ERα and ERβ on apical dendritic spines of CA1 pyramidal neurons compared to males, particularly during proestrus (Hyer et al., 2018; Shors et al., 2001; Woolley et al., 1990). This is of importance because estradiol modifies N-methyl-D-aspartate (NMDA) receptor signaling for driving sex-specific plasticity (Woolley and McEwen, 1994) and modulates hippocampal network cognitive activities (Raciti et al., 2023). In contrast to these findings, female rats experience a more pronounced reduction in dentate gyrus (DG) long-term potentiation (LTP) compared to males at 24 h and 7 days after TBI (White et al., 2017).

### Sex differences in animal models: general traits

Despite epidemiological data suggesting more favorable outcomes in males across measures of community integration (activities of daily living, return to work/school/sports, and Glasgow Outcome Scale), social-behavioral factors (cognitive function, depressive symptoms, memory, anxiety, and attention), and survival (Gupte et al., 2019), females often appear more resistant to TBI in experimental animal models. A scoping review found that 44% of animal TBI studies reported better outcomes in females, including measures of mortality, cognition, and sensorimotor function. In contrast, 14% of studies suggested worse outcomes in females, and the remainder reported no sex differences or mixed findings (Gupte et al., 2019). In models of repetitive mTBI, male rodents exhibited increased risk-taking and impulsive behavior, while females appeared relatively protected from these maladaptive changes but showed greater depressive-like behavior and anxiety (Krukowski et al., 2020; Wright et al., 2017). In models of msTBI, female mice showed reduced neuronal cell loss and improved motor outcomes compared to males, an effect that was attenuated by ovariectomy and associated with elevated post-TBI microgliosis and astrogliosis in hormone-depleted females (Clevenger et al., 2018). Together, these findings suggest that sex differences in TBI models are context-dependent, emerging across behavioral domains beyond memory. The reasons for the apparent contradiction between clinical and preclinical data remain unclear, but proposed contributors include differences in injury severity, symptom reporting and measurement, and the experimental models used. Notably, when psychosocial and other uncontrollable factors present in human TBI are minimized in the preclinical settings, estrogen and progesterone often emerge as protective.

## Memory pathophysiology

The sex-specific pathophysiological differences described above converge on memory circuits that are particularly vulnerable to disruption, shaping cognitive phenotypes after TBI. Understanding how hormonal modulation, neuroinflammation, and synaptic plasticity interact with injury-induced circuit disruption is essential for interpreting memory deficits.

Following msTBI, frontal and temporal structures, including the hippocampus, appear particularly vulnerable (Mioni et al., 2014). This is important because memory deficits in TBI patients are thought to arise, at least in part, from structural and physiological disruption of the hippocampus and associated circuits (Bohbot et al., 2000; Cave and Squire, 1991; Miller et al., 1998; Pierce et al., 1993; Reed and Squire, 1997; Rempel-Clower et al., 1996). Severe and fatal TBI can induce progressive hippocampal atrophy through cellular and synaptic loss, with particular vulnerability of neurons in CA3 (Baldwin et al., 1997; Chen et al., 1996; Golarai et al., 2001; Tang et al., 1997) and CA1 subfields (Bigler et al., 1996; Kotapka et al., 1992). Because these regions are central to learning and memory, hippocampal atrophy has been correlated with the severity of hippocampal-dependent memory impairment (Himanen et al., 2006; Moser et al., 1993; Tomaiuolo, 2004).

Following TBI, a depression in basal excitatory synaptic transmission in the CA1 subfield, as measured by excitatory post-synaptic potentials (EPSPs), has been documented (Miyazaki et al., 1992; Reeves et al., 2000; Zhang B. L. et al., 2011). This weaker baseline excitatory transmission is believed to hinder the CA1’s capacity to relay and amplify information from the CA3 region, resulting in diminished encoding of episodic memories. Additionally, increases in population spike amplitude and decreases in population spike thresholds have also been observed, indicating an alteration in the balance between inhibition and excitation (Miyazaki et al., 1992; Reeves et al., 1995, 1997; Witgen et al., 2005). This suggests a scenario in which pathological hyperexcitability may lead to less specific and more chaotic information processing. Conversely, an enhancement of basal EPSPs (Reeves et al., 1995; Santhakumar et al., 2001, 2000; Witgen et al., 2005; Zhang B. et al., 2011; Zhang B. L. et al., 2011) and depression of basal inhibitory postsynaptic currents (Bonislawski et al., 2007; Golarai et al., 2001; Hunt et al., 2011; Lowenstein et al., 1992; Mtchedlishvili et al., 2010; Witgen et al., 2005) have been reported in perforant path projections to the DG. As DG cells become more excitable and have disrupted filtering capacity (Lowenstein et al., 1992; Toth et al., 1997; Witgen et al., 2005), memory representations in CA3 and CA1 may overlap, causing difficulties in distinguishing similar experiences, contexts, and locations.

Compared to the hippocampus, less is known about post-TBI pathophysiological changes in the prefrontal cortex (PFC) (Smith et al., 2015). In animal models, a lower medial PFC excitability has been associated with working memory dysfunction (Smith et al., 2015). This may be mediated by shifts in intrinsic membrane properties (Yang et al., 2007), as well as morphological (Hoskison et al., 2009) and metabolic changes (Moro et al., 2011).

### Sex differences in memory in the clinic

Generally speaking, men appear to show better spatial memory, with an advantage in women in episodic and verbal memory (Maitland et al., 2004). Women recall their memories faster (Davis, 1999), date their memories more accurately (Skowronski et al., 1991), and use more emotional language when describing memories (Fuentes and Desrocher, 2013). Women also tend to surpass men in autobiographical memory (Fuentes and Desrocher, 2013), auditory episodic memory (Pauls et al., 2013), semantic memory (Maitland et al., 2004), random word recall (Herlitz et al., 1997), and facial recognition tasks (Herlitz and Yonker, 2002). Although men tend to perform better on spatial memory tasks, women are often superior in object-location memory tasks (Silverman and Choi, 2006). To date, relatively few studies have systematically examined sex differences in memory outcomes following TBI. However, existing evidence suggests that women may experience more pronounced memory impairments post-injury, particularly in domains such as episodic and recognition memory (Arachchi et al., 2026; Covassin and Bay, 2012; Howell and Griesbach, 2024).

## Preclinical memory deficits following TBI

As in clinical populations, relatively few preclinical studies have systematically examined sex differences in memory following TBI with most studies having historically used only male animals (Späni et al., 2018). Early work using moderate injury models, particularly the controlled cortical impact (CCI) and diffuse injury paradigms, largely reported no sex differences in memory performance (O’Connor et al., 2003; Wagner et al., 2007, 2004b; Xiong et al., 2007). However, more recent studies incorporating a wider range of injury severities, behavioral paradigms, and timepoints reveal a more nuanced picture, with sex differences emerging in a domain-specific manner (Table 1).

**TABLE 1:** Summary of preclinical studies examining sex differences in memory outcomes following traumatic brain injury.

Reference	TBI model; severity	Species	Memory test(s)	Main result
O’Connor et al., 2003	Impact-acceleration model of diffuse TBI; moderate	Rat	MWM	No sex difference
Wagner et al., 2004b	CCI; moderate	Rat	MWM	No sex difference. Estrous cycle and hormone level at time of injury has little effect on post-TBI behavioral performance.
Wagner et al., 2007	CCI; moderate	Rat	MWM, open field free novelty choice	No sex difference
Xiong et al., 2007	CCI; moderate	Mouse	MWM	No sex difference
Mychasiuk et al., 2014	Lateral CHI; mild	Rat	Novel context mismatch, social avoidance	No sex difference in novel context mismatch. More social avoidance behavior in females.
Free et al., 2017	CCI; moderate	Rat	MWM	No sex difference
Rubenstein et al., 2017	CHI; severe	Mouse	Active place avoidance and conflict avoidance	No sex difference
Semple et al., 2017	CCI; moderate-severe	Mouse	Social recognition	Fewer social recognition deficits in females
Wirth et al., 2017	Acceleration with impact; mild	Rat	MWM	Females show more persistent spatial memory deficits
Yamakawa et al., 2017	Repetitive CHI; mild	Rat	Elevated plus maze	Males have more deficits on elevated plus maze
Fitzgerald et al., 2022	Lateral FPI; mild-moderate	Mouse	PAL, BM, fear memory	Males were more vulnerable to anterograde memory deficits and appeared to show spatial learning impairment on BM
McCorkle et al., 2022	Repetitive CCI; mild	Rat	MWM, novel object recognition, novel object location	Males show earlier and broader deficits (1 week), while females exhibit delayed impairments (4 weeks). By 4 weeks, both sexes are impaired, but males show greater deficits in spatial tasks, with no remaining sex differences in object recognition.
McDonagh et al., 2025	Repetitive CHI; mild	Rat	BM	No sex differences
Santos Carlin et al., 2025	CCI; severe	Mice	MWM	Males show more impairment in cued and spatial learning. Females show sustained memory impairment.
Iannucci et al., 2026	Lateral FPI; mild	Mouse	Pattern recognition test	Females show more recognition memory impairment.

Summary of preclinical studies investigating sex differences in cognitive and memory outcomes following traumatic brain injury (TBI). Articles were identified through a literature search on PubMed using the keywords “sex,” “TBI” OR “brain injury,” and “cognition” OR “memory.” All relevant articles were screened for inclusion, and additional studies were identified through manual cross-reference searching of reference lists from selected articles. Included studies encompass a range of injury models, severities, and behavioral tasks assessing spatial, recognition, and social memory domains. BM, Barnes Maze; CCI, Controlled Cortical Impact; CHI, Closed Head Injury; FPI, Fluid Percussion Injury; MWM, Morris Water Maze; PAL, Paired Associate Learning.

### Interpreting sex differences in preclinical memory tasks

One factor that contributes to the difficulty in interpreting sex differences in TBI models involves the baseline sex differences in memory performance and strategy selection in animal studies. Males are often reported to outperform females in spatial memory tasks, potentially due to a greater reliance on allocentric and geometry-based “place” strategies, whereas females more frequently employ egocentric or landmark-based “cued” strategies (Fleischer and Frick, 2023). These preferences are not fixed and are influenced by circulating sex hormones, with both sexes shifting toward place-based strategies under high-hormone conditions. Additionally, differences in exploratory behavior, stress responsivity, and task engagement (such as thigmotactic or “serial” search strategies observed more commonly in females) (Locklear and Kritzer, 2014) can influence performance outcomes (Frick and Gresack, 2003; Hawley et al., 2013; Jonasson, 2005; Kanit et al., 2000; Koss et al., 2018; McConnell et al., 2012; Rodríguez Peris et al., 2024). Together, these inherent differences in cognitive strategy and baseline performance may mask or exaggerate injury-induced deficits, underscoring the need for careful interpretation and more standardized, sex-informed behavioral assessments in preclinical TBI research.

### Spatial memory

Spatial memory has been the most extensively studied domain in preclinical TBI research. This has been primarily assessed using the Morris Water Maze (MWM) and the Barnes Maze (BM). Early moderate CCI and diffuse injury studies in rodents largely revealed no sex differences in spatial memory performance (O’Connor et al., 2003; Wagner et al., 2007, 2004b; Xiong et al., 2007), a pattern that has been replicated in repetitive mild closed head injury (CHI) studies (McDonagh et al., 2025). In contrast, more recent reports suggest sex-dependent spatial memory trajectories. In mild injury rodent models, males tended to show earlier spatial memory impairment, while females exhibited delayed but persistent deficits – a pattern observed in both repetitive CCI and acceleration-impact paradigms (McCorkle et al., 2022; Wirth et al., 2017). Following mild-moderate lateral fluid percussion injury (FPI), males mice lagged in day-to-day transfer of spatial information on the BM, while retrograde spatial memory remained intact, suggesting a selective anterograde spatial memory acquisition vulnerability (Fitzgerald et al., 2022). Similarly, following severe CCI, male mice showed greater impairment in both cued and spatial learning on the MWM, while females showed greater impairment during probe trials, reflecting worse memory retention rather than learning *per se* (Santos Carlin et al., 2025). These findings suggest that, across a range of injury severities, males may be particularly vulnerable to disruptions in spatial learning acquisition, whereas female impairments may manifest more predominantly in memory consolidation and retention. Overall, spatial memory findings reveal sex- and severity-dependent patterns whereby males appear more vulnerable to acute and anterograde spatial learning impairments, while females tend to show delayed but persistent deficits and poorer retention. Several studies continue to report no sex differences in BM performance (Free et al., 2017; McDonagh et al., 2025), highlighting that task selection and injury model both influence whether sex differences emerge.

### Recognition memory

In animal studies, recognition memory has been assessed primarily through novel object recognition and pattern recognition tasks (PRT). In repetitive mild CCI, rats from both sexes showed recognition memory impairment, with no sex differences in object recognition observed at 4 weeks post-injury (McCorkle et al., 2022). In female mice with mild FPI, PRT performance (linked to hippocampal neurogenesis) was more severely impaired than in males at 35 days post-injury, suggesting greater female vulnerability in recognition-based memory (Iannucci et al., 2026). In a novel context mismatch task following lateral CHI, no sex differences were observed in rats (Mychasiuk et al., 2014). These results suggest that recognition memory findings are mixed, with female vulnerability emerging in some models but not others.

### Social and affective behavior

Social recognition memory appears relatively preserved in females after TBI. In moderate-severe CCI, female mice showed fewer social recognition deficits than males, suggesting a degree of female resilience in this domain (Semple et al., 2017). In contrast, affective outcomes are slightly different. Female rodents consistently show greater depressive-like behavior and increased anxiety following TBI, while males more commonly exhibit impulsive and risk-taking behavior (Wright et al., 2017). Greater social avoidance has also been observed in female rats following lateral CHI, likely reflecting increased stress reactivity than a memory deficit *per se* (Mychasiuk et al., 2014). Yet, other studies have shown worse performance in males on an elevated plus maze task (Yamakawa et al., 2017).

This pattern of greater female affective vulnerability after TBI may be mechanistically linked to sexually dimorphic HPA axis responses. Sex differences in hypothalamic neuroinflammation and hippocampal glucocorticoid receptor expression have been identified, with females showing patterns of changes consistent with a predisposition to late-onset HPA dysregulation (Bromberg et al., 2020). Given that glucocorticoid signaling in the hippocampus directly modulates memory consolidation, these findings provide a mechanistic bridge between HPA axis dysregulation, affective vulnerability, and persistent post-injury memory deficits observed in females. This is consistent with clinical evidence showing that affective symptoms after mTBI are more prevalent and severe in women than men (Bromberg et al., 2020), underscoring the importance of considering HPA axis functioning as a sex-specific therapeutic target in TBI recovery.

Fear-based memory tasks, though less frequently employed, provide insight into amygdala-dependent memory processing after injury. Following mild FPI in mice, cued fear retention was reduced in male mice, while females showed an opposing pattern (Fitzgerald et al., 2022). This dissociation between sexes suggests that amygdala-dependent memory circuits are differentially disrupted by TBI in males and females, and may interact with the sexually dimorphic HPA axis dysregulation described above to produce the divergent affective and mnemonic profiles seen across the literature.

### Limitations in translational validity

Despite the utility of rodent TBI preparations, their translational validity warrants consideration. Preclinical models demonstrate reasonable face validity, reproducing features of human TBI, including neuroinflammation, hippocampal pathology, and cognitive deficits. They also show construct validity, engaging overlapping mechanisms such as excitotoxicity, axonal injury, and blood-brain-barrier disruption. However, predictive validity remains limited, as therapies demonstrating efficacy in animal models are often not successfully translated to positive randomized controlled trials in humans. These limitations are particularly relevant when interpreting sex differences, as hormonal and neuroimmune factors that differ between sexes may interact differently with isolated versus complex injury. Additional sex-specific limitations include the frequent failure to document estrous cycle phase at the time of injury, the use of ovariectomized females, age-related hormonal variability that is rarely controlled for, and the potential for different injury models to preferentially detect male- or female-predominant deficits.

## Conclusion

Sex differences in post-TBI memory impairment are domain-specific and context-dependent, yet remain inconsistently characterized across clinical and preclinical research. Women generally experience worse cognitive and functional outcomes after TBI, while preclinical findings are more nuanced. Males tend toward earlier spatial learning impairments and anterograde deficits, whereas females more commonly show delayed consolidation and retention deficits alongside greater affective vulnerability. Neither sex shows uniformly worse outcomes across all domains or models.

These behavioral differences are supported by sexually dimorphic biological mechanisms, including hormonal modulation, neuroinflammatory trajectories, glucocorticoid receptor expression, and neuroplasticity patterns that have direct consequences for how therapeutic interventions may perform across sexes. Sex differences in drug metabolism, receptor expression, and pharmacodynamics are well established across neurological conditions (Sommer et al., 2024; Torrisi et al., 2026; Valentino et al., 2013), yet remain poorly characterized in TBI. Treatments targeting neuroinflammation, excitotoxicity, or HPA axis stabilization may require sex-stratified dosing or timing to achieve optimal outcomes, underscoring the importance of moving toward a precision medicine framework that treats sex as a stratification variable rather than a covariate to control for. Achieving this requires a fundamental shift in preclinical research practice. Inclusive designs incorporating both sexes, hormone status, estrous cycle phase, and a range of behavioral paradigms across multiple timepoints are necessary to capture the full complexity of sex differences in TBI. Standardization of reporting, including documentation of species, sex, injury model, and severity will be critical for meaningful cross-study comparisons.

Memory impairment remains one of the most debilitating and prevalent consequences of TBI, and a deeper understanding of how sex shapes its underlying mechanisms will be essential for developing targeted, sex-informed interventions that restore cognitive function and improve long-term outcomes.
